# Supplementary material for: Astrocytic CD44 Deficiency Reduces the Severity of Kainate-Induced Epilepsy
Source: Cells. 2023 May 26;12(11):1483. doi: 10.3390/cells12111483 (PMC10252631; doi:10.3390/cells12111483)
Supplement: Supplementary file 1 [file cells-12-01483-s001.zip › cells-2224795-supplementary.pdf]

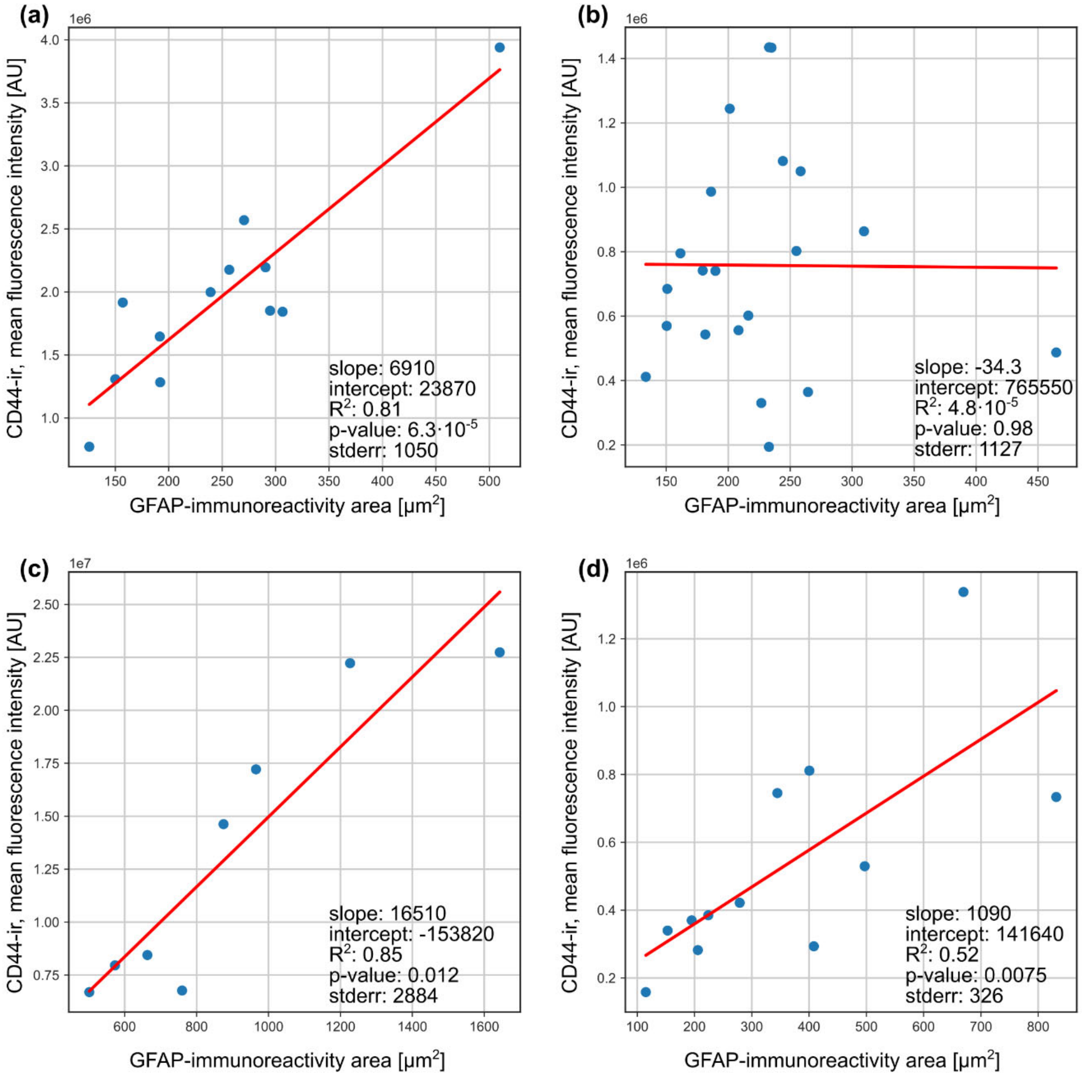

FigureS1. Correlation between the area of GFAP-ir and mean CD44 fluorescence for: (a): CTRL, (b) AsKO, (c) CTRL+KA, and (d) AsKO+KA groups. Statistical parameters in each panel correspond to the following parameters, respectively: slope and intercept of the fitted lines, R^2 (coefficient of determination), the p-value for a hypothesis test whose null hypothesis is that the slope is zero, standard error of the estimated slope. Lines represent linear regression given by the equation (GFAP-ir intensity (y) = slope∗ CD-44-ir intensity (x) + intercept).

Table S1

Fig 1c

	+SA	+KA
Shapiro-Wilk p-value:	0,33	0,15

power: 1.0

Fig 1d

	+SA	+KA
Shapiro-Wilk p-value:	0,06	0,23

power: 1.0

Fig 2c

	CTRL+KA	AsKO+KA
Shapiro-Wilk p-value:	0,63	0,20

power: 1.0

Fig 2d

	CTRL+KA	AsKO+KA
Shapiro-Wilk p-value:	0,75	0,06

power: 1.0

Fig 3b

	CTRL+KA	AsKO+KA
Shapiro-Wilk p-value:	<0.0001	<0.0001

power: 0.31

Fig 3c

	CTRL+KA	AsKO+KA
Shapiro-Wilk p-value:	0.89	0.59

power: 0.06

Fig 3d

	CTRL+KA	AsKO+KA
Shapiro-Wilk p-value:	0.15	0.06

power: 0.07

Fig 4c

	CTRL	AsKO
Shapiro-Wilk p-value:	0.07	0.002

power: 0.21

Fig 4d

	CTRL	AsKO
Shapiro-Wilk p-value:	0.14	0.48

power:1.0

Fig 4e

	CTRL+KA	AsKO+KA
Shapiro-Wilk p-value:	0.36	0.17

power:1.0

Fig 4f

	CTRL+KA	AsKO+KA
Shapiro-Wilk p-value:	0.09	0.06

power: 1.0

Fig 5c

	CTRL+KA	AsKO+KA
Shapiro-Wilk p-value:	0.21	0.78

power: 0.99

Fig 5f

	CTRL	AsKO	CTRL+KA	AsKO+KA
Shapiro-Wilk p-value:	0.18	0.71	0.44	0.78

power CTRL - CTRL+KA: 1.0

power CTRL - AsKO+KA:0.97

power AsKO - CTRL+KA:0.97

power CTRL+KA - AsKO+KA: 0.99

Fig 5g

	CTRL+KA	AsKO+KA
Shapiro-Wilk p-value:	<0.0001	<0.0001

power: 1.0

Fig 5h

	CTRL+KA	AsKO+KA
Shapiro-Wilk p-value:	<0.0001	<0.0001

power: 1.0

Table S1. Additional statistical parameters
